# Improving active participation during enterprise operations modeling with an extended story-card-method and participative modeling software

**DOI:** 10.1007/s10270-023-01083-8

**Published:** 2023-03-01

**Authors:** Marne De Vries, Petra Opperman

**Affiliations:** Department of Industrial and Systems Engineering, University of Pretoria, Pretoria, South Africa

**Keywords:** Participative enterprise modeling, Participative modeling software, Scaled agile, DEMO

## Abstract

The COVID-19 pandemic emphasized the need for process automation, using agile software development practices. However, when agile methods are used in *scaled contexts*, many software development efforts fail, mainly due to lacking requirements engineering practices. When business-oriented software needs to be developed within a scaled context, the *story-card method* (SCM), developed as part of a previous study, assists in structuring emerging software requirements within a taxonomy that represents enterprise operation. The SCM helps agile team members to develop a common understanding about enterprise operation when they construct the *enterprise operation taxonomy*. Digital *participatory enterprise modeling* (PEM) may increase collaboration and understanding among team members, especially when team members are geographically dispersed, when they co-model their understanding of enterprise operations. Using design science research to further evolve the existing SCM, we identified two concerns regarding the existing SCM: (1) The modeling software did not encourage *active participation during modeling*, and (2) Low quality of the resulting *cooperation structure diagram* (CSD) that is used to derive an *enterprise operation taxonomy*, i.e., the need to further extend the existing SCM. As main contribution of this article, we addressed previous deficiencies of the SCM, developing an *extended SCM* (eSCM), based on principles and guidelines that would encourage online participation during PEM, also providing a comprehensive case to demonstrate the eSCM. As a second contribution, we used survey-feedback from research participants, as well as activity tracking to evaluate whether the modeling tool encouraged active PEM. Our third contribution is to evaluate the quality of the resulting CSDs with suggestions for future improvement.

## Introduction

The COVID-19 pandemic emphasized the need for rapid adaption, driving process automation during the development or adaptation of supporting information systems. Although small-scale software development projects are well-supported by agile software development practices, additional requirements elicitation practices are needed to supplement agile software development methodologies for scaled contexts [1]. Agile teams, especially within scaled contexts, need to have shared mental models of software development goals [2], as well as a shared understanding of requirements [3]. Agile methodologies primarily use individual *user stories*, i.e., short user-oriented descriptions of software requirements [4], to package and release development work.

Since scaled agile projects need to create additional structure in allocating *user stories* to domains and sub-domains [5], the taxonomy of Forward & Lethbridge [6] is useful, providing a root level taxonomy for developing a particular type of software application. Since this paper presents an *extended story-card method* (eSCM) to structure user stories for a particular type of software application, the taxonomy of [6] is useful in positioning the eSCM. The root level of the taxonomy of [6], divides software into four main categories, based on the *dominance of a particular facet*: (A) Data-dominant software, (B) System-services software, (C) Control-dominant software, and (D) Computation-dominant software. The data-dominant software (root category A) has four categories, based on the target audience: (i) Consumer-oriented software, (ii) Business-oriented software, (iii) Design and engineering software, and (iv) Information display and transaction entry.

This study primarily focuses on the development of *business-oriented software*, i.e., software to support the daily *enterprise operations and their management*. Human beings need to interact regarding work activities that need to be performed. They need to share information on new production facts that come into existence, as well as the statuses of coordinating their activities. Within this operating context, information systems could semi-automate the coordination activities and facilitate information sharing. The Design and Engineering Methodology for Organizations (DEMO) *aspect models* are appropriate in specifying the *operating domain.* The operating domain could be further decomposed in *operating sub-domains*, each sub-domain requiring different domain expertise. As an example, the nature of operations within the manufacturing sub-domain is vastly different to on-boarding operations related to the post-graduate sub-domain.

Even though one of the DEMO aspect models, the coordination structure diagram (CSD), is useful reaching a *common understanding of enterprise operations*, prior in structuring emerging software requirements within a taxonomy of enterprise operations, previous research indicated that an additional story-card method (SCM) is required to systematically co-model existing enterprise operations in a consistent and concise way [7]. Multiple steps of the SCM can be synthesized into three main phases:

- *Phase 1* Task classification Applying steps 1–6, the modeling facilitator and the colleague map out some enterprise operations in the form of tasks, also called story-cards. They also classify the tasks, identifying tasks that are called original when the tasks produce new production facts versus informational when the tasks are used to share facts. Original tasks are color-coded in red or pink to distinguish between production acts versus coordination acts, respectively, whereas informational tasks are color-coded in green.
- *Phase 2* CSD modeling Applying steps 7–12 of the SCM, converts the color-coded story-cards into a diagram, called the coordination structure diagram (CSD). The SCM steps provide guidance to ensure that the enterprise operations are correctly depicted in terms of actor roles that interact with one another in coordinating their actions that are related to production of goods or services.
- *Phase 3* CSD validation The main purpose of this phase is to validate the completeness of the CSD. This phase is under-represented in the existing SCM and hence the eSCM introduced an additional step, i.e., Step 13, to validate the correctness and completeness of the CSD.

The problem with the existing SCM is two-fold: (1) The modeling software did not encourage *active participation during modeling*, and (2) Low quality of the resulting *cooperation structure diagram* (CSD) that is used to derive an *enterprise operation taxonomy*, i.e., the need to further extend the existing SCM.

The SCM is an existing artifact that needs further extension to address the two main concerns. Hence, our study falls within the Design Science Research Methodology (DSRM) genre according to the classification genres of design science research (DSR) identified by Peffers et al. [8]. Since a DSRM research effort may start in many different ways, also “with an already designed version of an artifact” [8, p 131], our study applies the DSRM genre of developing a new version of an artifact, namely an *extended* story-card method (eSCM).

Section 2 indicates how we applied the different phases of DSR to develop an *extended SCM* (eSCM). Section 3 provides background on the SCM as well as participative design (PD) and participative enterprise modeling (PEM). Based on previous concerns about the SCM, Sect. 4 presents an eSCM that incorporates a different modeling tool to encourage PEM. Section 5 presents results on experimenting with the eSCM, whereas Sect. 6 provides an additional discussion, reflecting on the limitations of the study and suggesting future work.

## Research methodology

Addressing the *five steps* of the DSR cycle, presented in [9], the eSCM was developed and evaluated.

### Identify a problem

A study that focused on the development and evaluation of an online-adapted version of the SCM [7], indicated two main concerns with the SCM: (1) The previous modeling software does not encourage *active participation* during modeling; and (2) The low quality of the resulting cooperation structure diagram (CSD) and hence its derived enterprise operation taxonomy, i.e., the need to further extend the existing SCM. Evaluating the *quality of the CDS* in the 2021-study, an average score of 57.4%, representing the level of understanding when applying Steps 7–12 of the SCM, indicated room for improvement. Further inspection of the resulting CSDs, also indicated that participants largely imitated a three-level hierarchy, as depicted in the SCM’s demonstration case. The latency of the previous modeling tool (Diagrams.net) is a problem, since there is a considerable delay in updating the diagram, which hampers *active participation* during modeling. One of the limitations of the survey that was used as part of the research methodology in the 2021-study, is that no evidence was extracted to confirm that a participant applied *interactive modeling* with the selected colleague, as required by the SCM. Informal feedback from some participants indicated that the latency problems of the tool *discouraged* interactive modeling.

### Define objectives of the solution

Four main objectives of the study are now discussed to address the two main concerns.

- *Objective 1* Select an online modeling tool that encourages participative modeling.
- *Objective 2* Develop an eSCM, applying principles from participative enterprise modeling (PEM), and present a more comprehensive case to demonstrate the eSCM.
- *Objective 3* Evaluate whether the selected tool *encourages participative modeling* and evaluate the *level of participation* during interactive modeling.
- *Objective 4* Improve the quality of the resulting CSD, by adding more guidance for Steps 7–12 of the SCM, adding Step 13 (to compile a *transactor product table*) to further validate the CSD, and clarifying conditions for using a deep versus a flat hierarchical structure. The need for Step 13 is linked to the results of [7], indicating that the existing SCM produced low-quality CSDs. The *transactor product table* is introduced as an additional mechanism to validate the CSD.

### Design and development

Addressing *Objective 1*, we selected an appropriate modeling tool by experimenting with 2 competing tools, used in combination with the eSCM, involving 2 research participants during the experimental phase (see Sect. 4.1). Addressing *Objective 2*, we developed the eSCM, as well as a demonstration case that is based on a post-graduate operating context at a fictitious tertiary education institution, ensuring ease-of-understanding for the *research participants* with different academic and industry contexts (see Sect. 4).

### Demonstration

The eSCM was demonstrated to industry participants during an interactive online session, using the comprehensive post-graduate case. During the demonstration, participants had the opportunity to criticize the *method*. The feedback was also used to further refine the eSCM so that participants could apply the same eSCM that is presented in this article (see Sect. 4.1).

### Evaluation

Each of the 36 research participants applied the eSCM in practice by involving a colleague from industry. Addressing *Objective 3*, we used a survey of 27 items (questions and probes), voluntarily completed by 25 participants (see Sect. 5.3). The survey, evaluated whether the selected modeling tool *encouraged participative modeling*. Since the new modeling tool provided a feature of tracking the modeling actions of participants, we also report on the *level of participation* when participants applied the participation instructions embedded in the eSCM. For *Objective 4*, we used survey-feedback on the changes that were incorporated for the eSCM to improve the quality of the diagrams (see Sect. 5.2), and also evaluated the diagrams that were submitted by the 36 participants (see Sect. 5.4).

## Background and related work

According to Alhazmi & Huang [10] regular user stories do not explicitly support requirements traceability in software development, which causes problems when requirements, code and tests are changed over time. For scaled agile projects, additional practices are needed to structure and trace emerging requirements [11, 12]. When new software has to support the day-to-day operations of an enterprise, the software development team should have a common understanding of the operating context. Dietz and Mulder [13] present four ontological aspect models that are coherent, comprehensive, consistent, and concise and that are useful to represent the essence of enterprise operation [13]. The Cooperation Model (CM), consisting of two representations, the Coordination Structure Diagram (CSD) and the Transactor Product Table (TPT) [13], can be used to structure some of the emerging software requirements [7]. Since the CSD concepts are very abstract, an additional story-card method (SCM) is needed to facilitate the participative construction of the CM for a particular scope of enterprise operations.

Using the post-graduate sub-domain as a fictitious case, post-graduate operations involve primary activities, such as *proposal evaluating*, *admitting* and *registering* activities. Elaborating on these three activities, post-graduate students that enroll at the fictitious enterprise, i.e., the tertiary education institution, need to first submit a proposal that is linked to a particular focus area, such *as climate change.* The *proposal has to be evaluated* by a possible supervisor of the proposed study. Once the proposed study has been approved, the student has to be *admitted* to a post-graduate study program. A condition for finalizing admission to the study program, is that the student needs to *register* for the study program, also paying a registration fee.

Human beings involved with post-graduate activities, coordinate their acts around these primary activities. The primary activities are called *elementary transaction kinds*, in accordance with the PSI (performance in social interaction) theory presented in Dietz and Mulder [13]. Since each elementary transaction kind (e.g., proposal evaluating) is executed by only one elementary transactor role (e.g., proposal evaluator), the elementary transactor roles become useful as a taxonomy, structuring user stories that may emerge as new requirements for a supporting information system. The elementary chunks of elementary transactor roles are particularly useful to frame user stories, using a consistent set of roles throughout an agile software development project. Identifying the elementary transactor roles and how these roles coordinate with one another, agile team members need to have thorough knowledge about DEMO and its foundational theories. A graphical representation of the interacting transactor roles, using a Cooperation Model (CM), provides a common understanding, to agile team members, about those enterprise operations that need to be semi-automated. Yet, the CM’s representations, i.e., the cooperation structure diagram (CSD) and transactor product table (TPT), are not as intuitive, when compared to other enterprise operation diagrams, such as collaboration diagrams expressed in Business Process Modeling Notation (BPMN) [14].

An additional method, called the *story-card method* (SCM) is useful to fast track training, when the DEMO-trained facilitator systematically imparts knowledge about cooperation modeling to an agile team, while co-developing a CSD to create a common understanding about the operations of a particular enterprise [7]. An experimental study, applying the SCM, indicated that research participants were positive, indicating that the SCM facilitated collaboration and translation of concrete concepts into more abstract (and concise) concepts of the CSD [7].

In Sect. 3.1, we provide more background on the CM and how it depicts the essence of enterprise operation. Section 3.3 provides literature on the existing knowledge area of participative enterprise modeling and Sect. 3.6 extracts criteria for effective participative enterprise modeling. Based on our literature review on participative enterprise modeling, we motivate our tool selection for the eSCM, in Sect. 4.1.

### The cooperation model

According to Bouling’s [15], hierarchy of complexities, enterprises as socio-cultural systems, are positioned as level-8 entities on a 9-level complexity scale. One way of dealing with complexity is to hide complexity, assisting the human ability to comprehend complex entities [13]. Based on three enterprise engineering theories, the PSI (performance in social interaction) theory, ALPHA (abstraction layers in production for holistic analysis) theory and the OMEGA (organizational modules emerging from general arrangements) theory, the Cooperation Model (CM) reduces the perceived enterprise complexity by representing the *essence of enterprise operation* [13].

Since we need to explain some of the theoretical content, we introduce a fictitious case, i.e., post-graduate operating context, to explain theory about the CM. The post-graduate operating context include some post-graduate operations at a tertiary education institution. Some of the primary activities of the post-graduate operations include proposal evaluating, admitting and registering activities.

In terms of the *PSI theory*, the CM acknowledges that enterprise operations can be perceived as actor roles that perform multiple coordination acts and production acts. The coordination acts and production acts are performed in a particular sequence, called transaction patterns. Instead of mapping out the detailed transaction patterns for each *kind of transaction* that takes place, the CM hides the detail of these consistent transaction patterns between two actor roles, hiding some of the complexity to enable human understanding. Therefore, The CM will extract the different *kinds of transactions* (e.g., proposal evaluating, admitting and registering), hiding the detailed coordination activities that form part of these transaction kinds.

In terms of the *ALPHA theory*, the CM abstracts from technological implementation and realization detail, only representing *original* transaction kinds, excluding the informational and documental transaction kinds [13]. Using the post-graduate context, the CM will include the original transaction kind *proposal evaluating*, but it will *exclude* its supporting transaction kinds to hide complexity. Even though *proposal evaluating* should be supported by informational transaction kinds, such as *student undergraduate qualification sharing*, and documental transaction kinds, such as *proposal retrieving*, the CM will only include *proposal evaluating* as an original transaction kind.

In terms of the *OMEGA theory*, the CM indicates that three different structures guide the operations of an enterprise, namely the *interaction structure*, *interstriction structure* and *interimpediment structure*. We will elaborate more on these structures when we discuss the CSD in more detail.

**Fig. 1:**
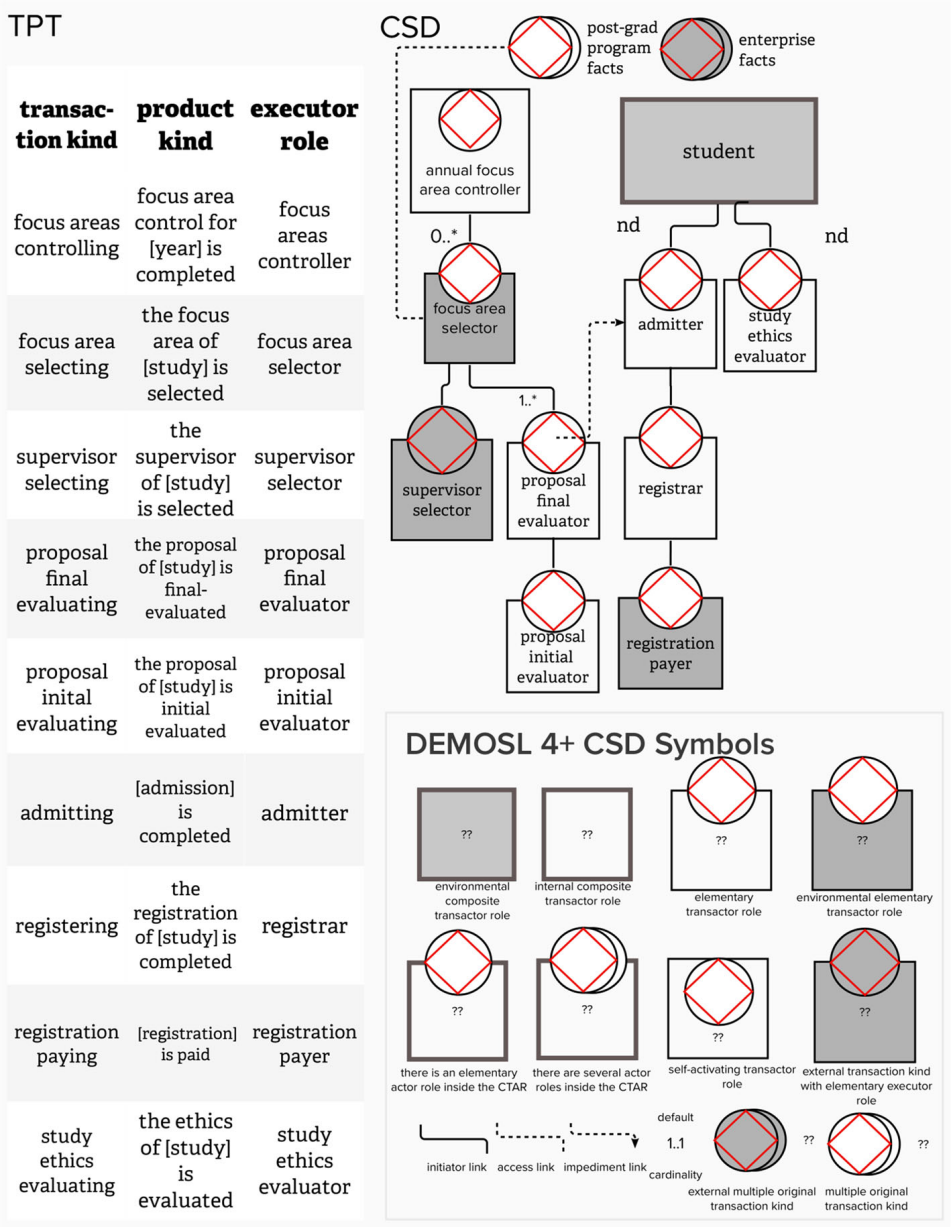
CSD and TPT modeled with MURAL

The CM can be represented by a Coordination Structure Diagram (CSD) and Transactor Product Table (TPT). In discussing the main constructs of a CSD, the right-hand side of Fig. 1 provides a graphical representation of a CSD that consists of nine transactor roles. Each of the *elementary transactor roles* include two components, an actor role (quadrilateral) and a transaction kind (diamond-disc). The description of the transaction kind is provided on the TPT, indicated on the left-hand side of Fig. 1. Thus, the transactor role named *proposal evaluator*, indicates that the actor role *proposal evaluator* is the executor of a single transaction kind, called *proposal evaluating*. The transactor role is a representation of a complete transaction pattern that exists when an initiator starts the transaction pattern for the transaction kind.

The CSD and TPT presented in Fig. 1, provide a concise representation of a fictitious enterprise that offers *some* post-graduate operations at a tertiary education institution. The analyst (and software development team) has to decide on a scope-of-interest (SoI), i.e., *some* (not all) enterprise operations that need to be supported by software. For our fictitious case, we selected *some* post-graduate operations of the fictitious enterprise. Based on the selected SoI, all white quadrilaterals indicate human actor roles that are considered to be *inside* the selected SoI (e.g., admitter), whereas gray-shaded quadrilaterals with a white diamond-disk indicate human beings that are within the direct *environment* (e.g., registration payer), and gray-shaded quadrilaterals with a gray diamond-disk are external to the SoI (e.g., supervisor selector). The SoI is further demarcated by environmental composite actor roles, indicated by thick-bordered gray-shaded quadrilaterals (e.g., student) that only act as initiators for the selected SoI.

The CSD, in accordance with the OMEGA theory, highlights three different kinds of *coordination structures*, including the (1) interaction structure, (2) interstriction structure, and the (3) interimpediment structure [13]. Partially explaining Fig. 1 as a representation of *some* post-graduate operations, demonstrating the three coordination structures, we use *italics* style when we refer to a construct in Fig. 1. The legend for constructs included in Fig. 1, is shown on the right-hand side, in accordance with [13].

#### Interaction structure

The first type of link displayed in Fig. 1 is the initiator link, represented by a solid line between constructs. As an example, a *student* initiates interaction with an *admitter*. The *student* also initiates interaction with the *study ethics evaluator*.

Some of the initiation links in Fig. 1 also include annotations that indicate the minimum and maximum number of initiations that could be created. For instance, the initiation link between *focus area selector* and *proposal final evaluator* indicates “1.*” as the annotation. The implication is that a *focus area selector* can initiate a minimum of *one* and a maximum of *many* instances of *proposal final evaluating*. Motivating this cardinality, the *focus area selector* may offer different study proposals as possible studies within a selected focus area, hoping that one of these proposals will be approved.

#### Interstriction structure

A second type of link exists in Fig. 1, namely the access link, represented by a dotted line, with *no* arrow-head. The access link implies access to certain facts. The dotted line between the *focus area selector* and *post-grad program facts* indicate that the *focus area selector* needs to have access to facts that have been created already. Access to these facts are necessary, since they *restri*ct the operating behavior of the *focus area selector*.

#### Interimpediment structure

A third type of link exists in Fig. 1, namely the wait link, represented by a dotted line *with* arrow-head. The wait link indicates that the progress of a particular actor role, responsible for acts related to an instance of a transaction kind, may be impeded by the progress of acts regarding *other* transaction kind instances. The single wait link in Fig. 1 indicates that progress regarding a *proposal final evaluation* instance impedes the *admitter’s* progress on an *admission* instance.

### The story-card method

As indicated in Sect. 1, user stories are concise descriptions of software requirements, useful to package and release development work for agile software development projects. Yet, scaled agile projects need to create additional structure in allocating user stories to domains and sub-domains [5]. The CM is appropriate to specify the operating domain of an enterprise and provide a starting point for structuring user stories that relate to the operating domain [16]. Although the CM has a strong theoretical foundation and the means to hide complexity in a consistent way, extensive training is needed, since the underlying concepts associated with DEMOSL (Design and Engineering Methodology for Organization Specification Language) are not as intuitive when compared to other languages, such as BPMN, in representing the operating domain [14]. A DEMOSL 3-based Story-card Method (SCM) was suggested in 2018 as a means to incorporate one of the DEMO diagrams into scaled agile methodologies [16].

The SCM was constructed to link *user stories* to a *big picture* representation of the operating context, consisting of ten steps. Feedback from participants that applied the SCM were positive [16] and the SCM was applied to a real-world project [17]. Yet, due to the COVID-19 pandemic, the face-to-face and sticky note format of the SCM had to be converted to an online platform, using Diagrams.net as a modeling tool and adapting the SCM to accommodate the new DEMOSL 4 + version [7]. Although participants were positive, the quality of the CSD’s were still problematic. In addition, the modeling software (Diagrams.net) did not encourage *active participation* to gain a common understanding of the operating domain [7]. One of the main objectives of using the SCM is that the agile team members need to participate in co-modeling the operating context. The resulting CSD, has to provide a common understanding of enterprise operation before it is used as an *enterprise operation taxonomy* to structure emerging user stories. Prior to the further development of an eSCM, we had to answer a key question: *What can we learn from existing knowledge areas, such as participative design and participative enterprise modeling to guide the development of the eSCM?*

### Participative design and participative enterprise modeling

Two knowledge areas developed in parallel, both sharing a participative approach, namely *participative design* and *participative enterprise modeling*.

Participative design (PD) emerged as a method within human–computer interaction (HCI) and software design for more than a decade [18]. The main objective of using participatory design is to make the consumers as end-users, *part* of the design process, rather than involving the consumers right at the end of the design [19]. According to Simonsen and Roberson [20], participatory design supports mutual learning between multiple participants in collective “reflection-in-action.” Since PD is used when designing a new artifact, an entire design cycle may be implied, starting with an existing understanding of a current artifact or process that needs to be re-designed, also including participation in selecting among different choices for solution areas and solution constructs [21].

Enterprise modeling (EM) is “an integrated and multi-perspective way of capturing and analyzing enterprise solutions” [22, p 1]. Enterprise models may be created to serve different objectives, also as part of a design cycle to (re-)design a part of the enterprise. When multiple individuals are involved during modeling, EM can be further classified as collaborative or participatory, most effectively conducted when a facilitator leads the collaborative modeling session [22]. Fellman et al. [23] indicate that *collaborative* modeling emphasizes joining of *several experts* into a coordinated effort, whereas *participative* enterprise modeling (PEM) also involve *users or enterprise stakeholders*. One of the main objectives of a participative approach for EM is avoiding conceptual misalignment between the stakeholders and their different perspectives [23]. PEM is also aligned with the paradigm of the 2018 BISE research note, moving enterprise modeling from an expert discipline toward a more inclusive modeling approach [24]. In guiding our participative approach when we extend the SCM, we extract guidance from both knowledge areas, presented in the following two sections.

### Principles and approaches for participative design

Bratteteig and Wagner [21] indicate that both *influence*, as well as *context* shape the level of participation during PD. Structural elements of the project context may limit the possibilities to participate and make choices. When users participate in the design process, the design process itself may still diminish their voice in and influence on the future design of an enterprise artifact. Some of the core principles of PD include participation and democracy [25] equalizing power relations [26] and imply value-centered design, since an ethical stand has to be taken to recognize accountability when designing a world and the lives of those inhabiting the newly-created world [27]. Reynolds and Hansen [28] believe that a key condition for PD is that participants need to be willing to learn. Since the involved stakeholders have different backgrounds and experiences, they need to be respectful about alternative visions about technology [26].

Hansen et al. [19] identified two main approaches that may be applied during PD, namely a linear approach or a nonlinear approach. The linear approach is characterized by sequential phases, exemplified by Akoglu and Dankl’s [29], four phases of (1) meeting with stakeholders, (2) switching over roles, (3) voice ideas, and (4) evaluation. The nonlinear approach reflects the iterative nature of design projects and participation that is represented as iterative cycles. An example of such an approach, is the nonlinear four-staged approach of Sanders and Stappers [30] that consists of (1) pre-design, (2) generative, (3) evaluative, and (4) post-design stages, where different methods, tools and techniques may be used within a PD session [31].

### Guidelines of participative enterprise modeling

The Participative Enterprise Modeling (PEM) body-of-knowledge also provide principles and practical guidelines to facilitate participation among stakeholders. PEM requires modeling sessions that need dedicated persons who know how to organize a modeling project, the modeling sessions and the aspects that influence the success and efficiency of the modeling practice. Stirna and Persson [22] emphasize that analyst-driven models often lack important aspects and details of the organization when relevant participants are not actively involved during enterprise modeling and design [22]. Highlighting three main characteristics, Stirna and Persson [22] indicate that a participative approach has: (1) a defined way of working in the form of methodological steps to carry out the modeling sessions with explicit principles of stakeholder involvement; (2) a group of stakeholders responsible for the knowledge that goes into the model; and (3) a modeling facilitator responsible for guiding the discussion among stakeholders and the modeling method used.

Gutschmidt et al. [32] already identified a number of authors that defined *patterns* for human–computer interaction, encouraging participative modeling, with the intent of identifying requirements of a multi-touch-table tool. Even though the multi-touch-table is a *physical table*, allowing participants to provide different perspectives when the users are “standing at all sides of the table” [32, p 4], the *patterns* may also be useful when *online participative modeling tools* are designed or selected for online participative enterprise modeling. Some of these patterns include: (1) *Hovering functions* for mouse-based applications, where elements are only displayed when a mouse icon hovers over an object, optimizing the use of workspace; (2) *Zooming functions* to support visual reachability; and (3) *User identification* to support balanced participation.

### Structuring for participative modeling

Within the field of PEM, Fellman et al. [23] identified the need to provide more advice on how to structure participative enterprise modeling sessions. Drawing from two real-world cases where PEM was applied, they derived a generic workshop process model that includes three main phases: (1) Preparation; (2) Execution; and (3) Finalization [23]. Each of the two real-world cases incorporated a workshop with multiple participants, i.e., 10 participants in Case A (modeling the digital transformation goals at an automotive supplier) and 10 participants in Case B (modeling the innovation process at a manufacturing company). For our study, reported in this article, we experimented with participant-pairs, rather than large workshops and therefore it is possible that a simpler process would suffice. Therefore, we only used the three-phased process as a *guideline* to structure the participant-pair sessions, focusing on the *execution* phase. Since our participative session had to be conducted online, we had to select a modeling tool that would allow and encourage participation during co-modeling. We had to evaluate existing tools, selecting an appropriate tool that would encourage active participation.

### Related work on tool evaluation

Other research scholars within the knowledge area of PEM have already experimented with online PEM tools. Gutschmidt [33] experimented with two freely-available tools, namely Draw.io and Google Drawings. As indicated in Sect. 1, the previous version of the SCM also suggested Diagrams.net (a new branding for Draw.io), highlighting the latency problems with Diagrams.net [7]. The study of Gutschmidt [33] was useful in extracting some of the features of online PEM tools that contribute toward a positive *perception* of PEM tooling. Using the technology acceptance model (TAM) model [33] as a basis, Gutschmidt [33] measured the perception of PEM tooling in terms of five *perception* criteria: (1) perceived usefulness, (2) perceived ease-of-use, (3) perceived enjoyment, (4) acceptance, and (5) awareness (of changes made by another participant).

Gutschmidt [33] invited four teams of three students to create two different kind of models, a goal model and process model, where each team had to use a *single* tool, either Google Drawings or Draw.io. Furthermore, the teams used Zoom for communication. Due to the small sample size, factor analysis and significance tests were excluded. Overall, Draw.io scored better than Google Drawings for both goal modeling and process modeling, in terms of most of the five perception criteria. The interviews were analyzed to extract positive and negative aspects of the modeling tool. Even though Draw.io was selected as the superior tool, some negative aspects were highlighted. Some of these negative aspects have already been addressed by other free-to-use tools that we included in our sample of digital PEM tools.

As indicated in the next section, we used a different approach to experiment with multiple tools, with the main objective of selecting a single tool for our main experiment.

## The extended story-card method (eSCM)

Addressing *Objective 2*, we developed the eSCM as a template on MURAL in accordance with the principles, participation guidelines and session structuring given in Sects. 3.4, 3.5 and 3.6. Section 4.1 provides detail about the content of the eSCM as well as the resulting diagrams that are based on the post-graduate demonstration case. Section 4.3 presents the implementation of the eSCM using MURAL.

### Tool experimentation and comparison

In addressing *Objective 1* of this study, indicated in Sect. 2, we used an iterative process to experiment with two participative modeling tools, namely Miro and MURAL. One of the key entry requirements for selecting a tool as a candidate was ease-of-modeling. The two main researchers had full administrative rights on the tools and used an exploratory or inductive approach to identify the main features of the tools. In addition, the two main researchers experimented with some of the existing flow-charting templates offered by the two tools to assess the ease-of-modeling. The experimental process consisted of three main phases producing tool comparison results, synthesized in Table 1: (1) *feature exploration*, (2) *entry requirement identification*, and (3) *evaluation*.

**Table 1:**
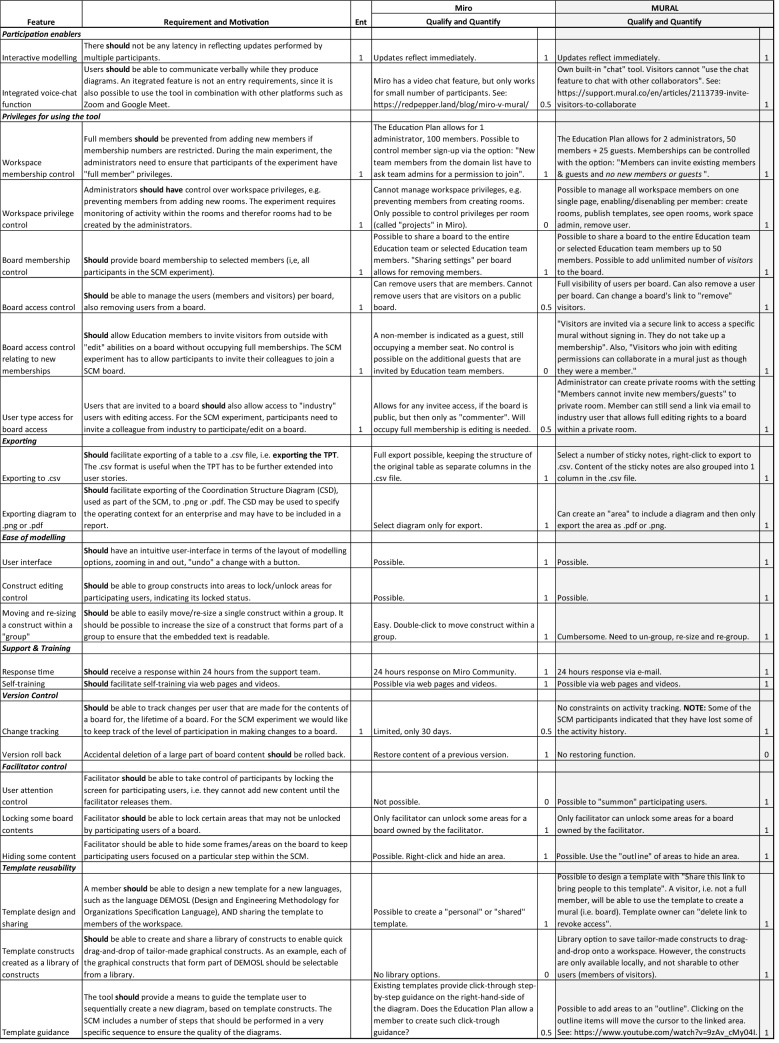
Tool comparison results

Since the two main researchers also had to evaluate the participative abilities of the tools for tool users that do not have administrative rights, two additional participants formed part of the experimentation team and were involved to act as *members* or *visitors* on the tooling platforms, with restricted access rights. The two additional participants were only involved during the *feature exploration* phase.

During the *feature exploration* phase, the two main researchers collaboratively elicited tool requirements, using a shared Excel spreadsheet to capture new requirements as they emerged.

- Using a deductive approach, building onto existing knowledge about the previous modeling tool (Diagrams.net) that was used in combination with the SCM in [7], tool features had to answer the question: *What features are needed, when used in combination with the SCM, encouraging participative modeling?* One of the researchers experimented with two no-cost platforms, offered via their Education Plan, developing a SCM template for both Miro and MURAL, ensuring that an easy-to-use template could be developed.
- Using an inductive approach, the two main researchers experimented with the two no-cost platforms to answer the question: *While experimenting with the tool, what existing features encourage participation and promote ease-of-use?*
- Table 1 provides a summary of the tool features. The first column of Table 1, *Feature*, also provide sub-headings (indicated in bold and italics) to group features into themes. Next to *Features*, the column *Requirement and Motivation* provides a more detailed description of the SCM-related requirement in terms of the indicated *Feature*.

During the *entry requirement identification* phase, the two main researchers identified those features that are absolutely necessary, when used in combination with the SCM. The column *Ent* in Table 1 indicates with a “1” whether a particular feature has been classified as an entry requirement.

During the *evaluation* phase, we used two columns in Table 1 to record the evaluation results, i.e., *Miro* and *MURAL*. For each of the tools we *Qualify* our evaluation results, providing a description on how the tool addresses the corresponding requirement. In addition, we *Quantify* the level of addressing a requirement as 1 (fully addressed), 0.5 (partially addressed) and 0 (not addressed).

Using only the *entry requirements* when comparing the tools, Miro is disqualified, for not addressing all of the *entry requirements*. Yet, we included our full analysis in Table 1, since participative modeling tools are still developing and we acknowledge that MURAL may not be the best tool to use when other researchers repeat the comparison in future. We also believe that the inductive exploration of features may be useful to other researchers when a different set of entry criteria apply with their specific participative modeling context, acknowledging that the requirements stipulated in Table 1 are focused on supporting *online/real-time cloud-based participative modeling* when team members work remotely. Unfortunately, online participative modeling tools lack many features that repository-based tools offer, such as configuration control, automatic model validation, and even model transformation (see [34]).

A third no-cost platform was also discovered later in the study, called *FigJam*. One of the entry requirements presented in Table 1, relate to the feature “User type access for board access,” indicate that “Users that are invited to a board, should also allow access to "industry" users with editing access.” For FigJam, users with editing rights need to be registered students. Since our experiment with the SCM involved industry participants, we excluded *FigJam* as an option.

### The eSCM content

The main objective of the eSCM is to reach a common understanding about some enterprise operations when the participants co-model an *enterprise operation taxonomy*, represented by the coordination structure diagram (CSD). The eSCM deviates from participative enterprise modeling norm where the norm indicates that training should be avoided. Even though the first step encourages inputs from the non-facilitator to model an existing process, using simple flow-charting that requires no or little training, the purpose of the eSCM is *also* to transfer knowledge on systematically converting flow-charting knowledge into a CSD that depicts and enterprise operation taxonomy.

As indicated in Sect. 2.2, *Objective 4* of this study is about improving the quality of the resulting CSD, by adding more guidance for Steps 7–12 of the SCM, adding Step 13 (to compile a *transactor product table*) to further validate the CSD, and clarifying conditions for using a deep versus a flat hierarchical structure. Comparing the SCM (presented in [7]) with the eSCM, we now present the eSCM in the first column of Table 2, gray-shading the content that were changed or added in the eSCM when compared to the SCM. More detail about the nature of the change is indicated in the second column of Table 2.

**Table 2:**
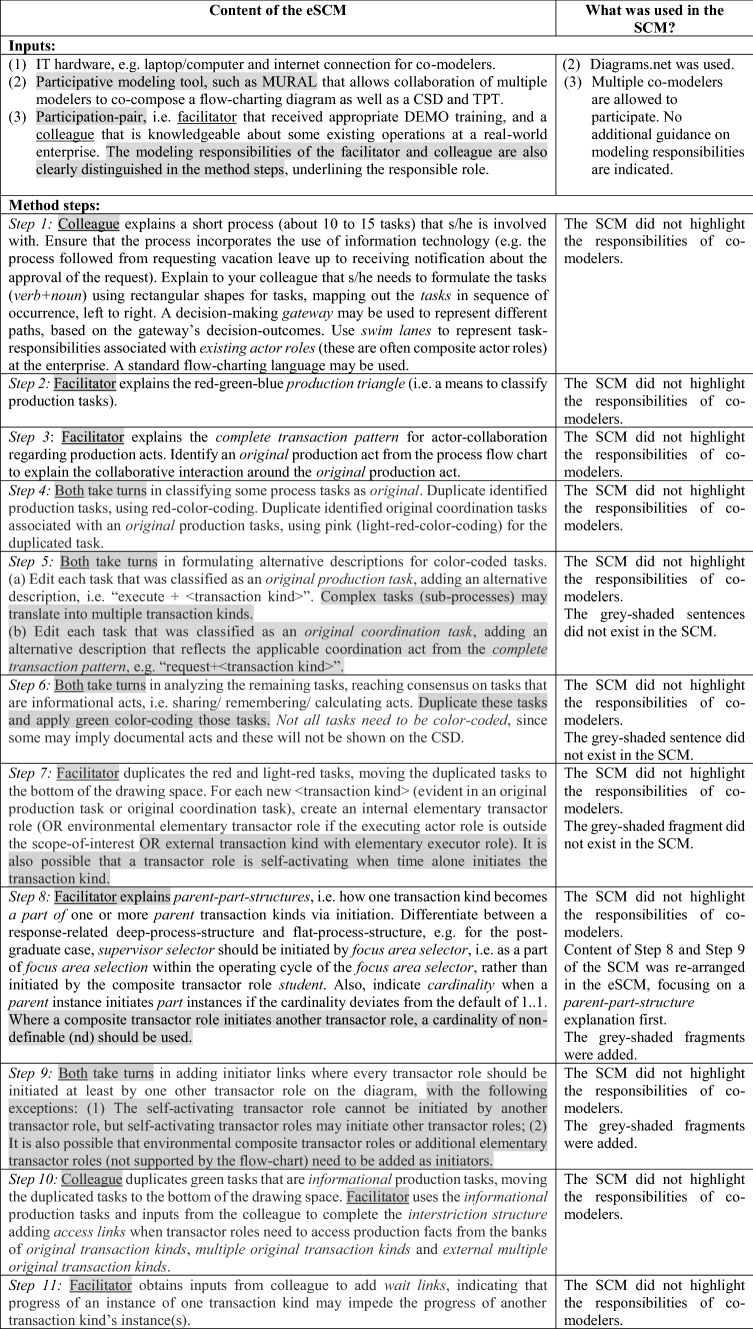

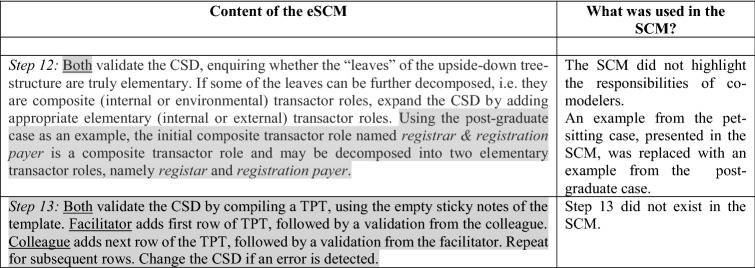
Content of the eSCM compared to the SCM

The eSCM specifies 3 inputs and 13 method steps.

**Fig. 2:**
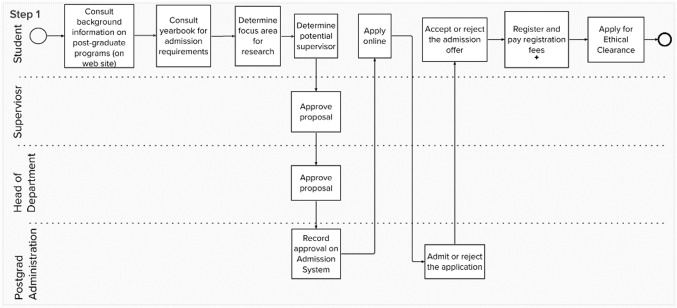
Example of a post-graduate case step 1 of the SCM

**Fig. 3:**
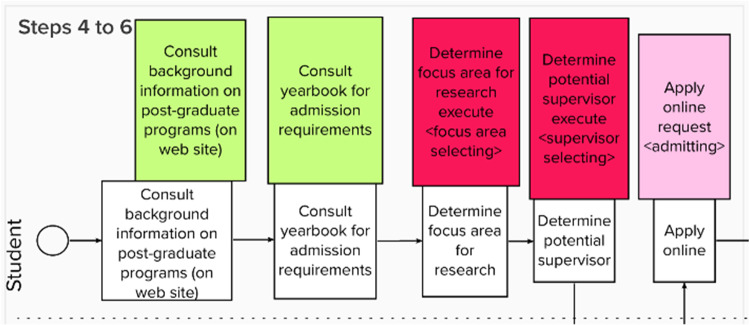
Extract of analyzing the flowchart, incorporating steps 4 to 6 of the SCM

**Fig. 4:**
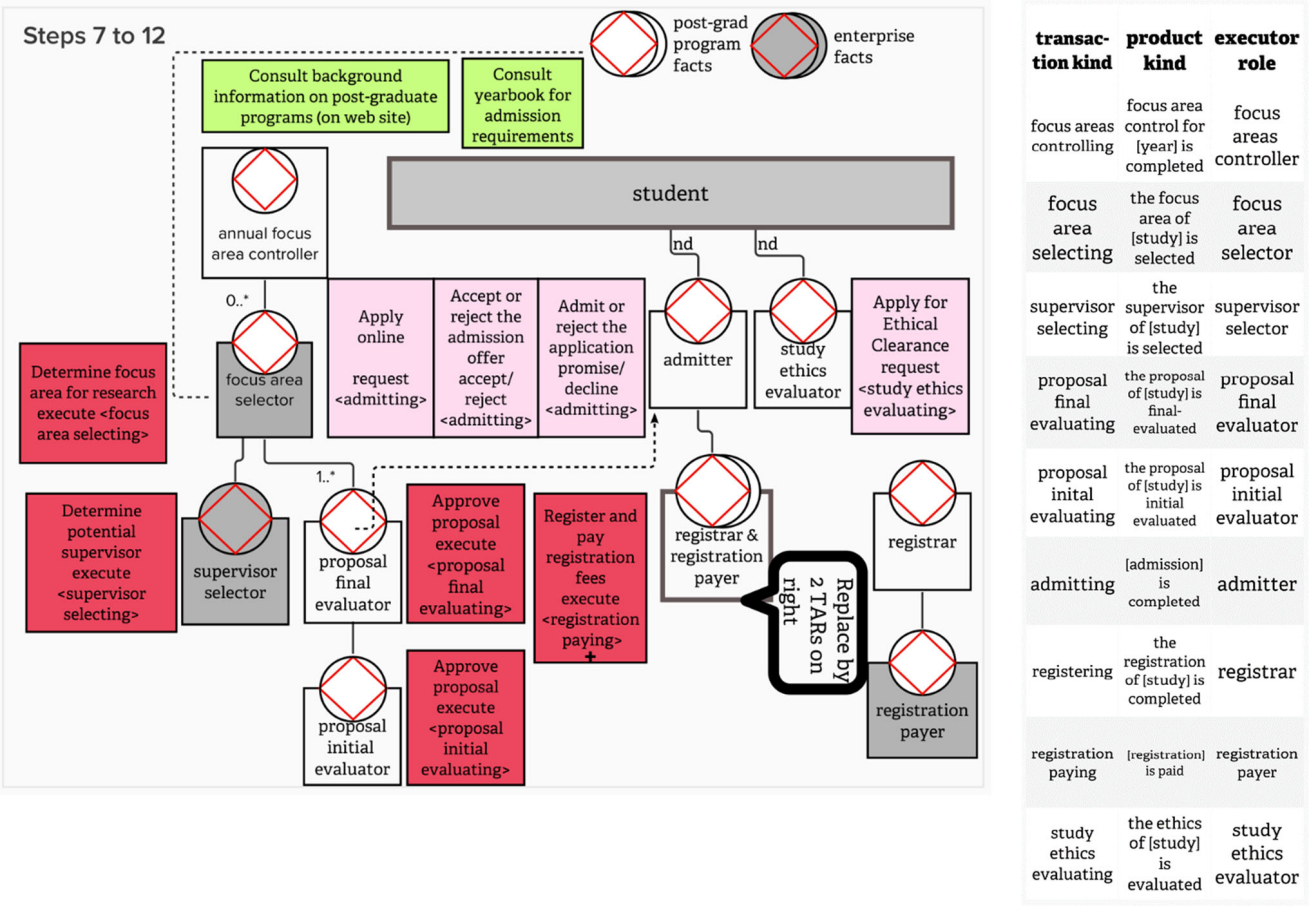
Constructing a CSD for Steps 7 to 12 of the SCM (left-hand side) and compiling a TPT during step 13 of the SCM (right-hand side)

The method steps were demonstrated to the participants, based on the post-graduate case, starting with a flowchart of some operations at a fictitious tertiary education institution. Figure 2 represents the flowchart, i.e., the result of performing *Step 1* of the eSCM. Since *Steps 2 and 3*, are explanation steps, no graphical representations are included for these steps. Figure 3 results from performing *Steps 4 to 6* and Fig. 4 results from performing Steps 7–13.

Elaborating on how Steps 7–12 assisted in shaping the CSD that is shown in the left-hand side of Fig. 4, Table 3 provides a repetition of the eSCM (Steps 7–12) in the first column, providing an extract from Fig. 4 in the second column.

**Table 3:** Applying steps 7 to 12 of the eSCM

Content of the eSCM	Extract from Fig. 4 explaining each step	
*Step 7:* Facilitator duplicates the red and light-red tasks, moving the duplicated tasks to the bottom of the drawing space. For each new < transaction kind > (evident in an original production task or original coordination task), create an internal elementary transactor role (OR environmental elementary transactor role if the executing actor role is outside the scope-of-interest OR external transaction kind with elementary executor role). It is also possible that a transactor role is self-activating when time alone initiates the transaction kind	In the extract below, the focus area selector is an environmental elementary transactor role, since the student (with role *focus area selector*) is not employed by the fictitious enterprise. The *proposal final evaluator* is internal elementary, since *proposal final evaluator* is inside the scope-of-interest, i.e., employed by the enterprise	
*Step 8:* Facilitator explains *parent-part-structures*, i.e., how one transaction kind becomes *a part of* one or more *parent* transaction kinds via initiation. Differentiate between a response-related deep-process-structure and flat-process-structure, e.g., for the post-graduate case, *supervisor selector* should be initiated by *focus area selector*, i.e., as a part of *focus area selection* within the operating cycle of the *focus area selector*, rather than initiated by the composite transactor role *student*. Also, indicate *cardinality* when a *parent* instance initiates *part* instances if the cardinality deviates from the default of 1..1. Where a composite transactor role initiates another transactor role, a cardinality of non-definable (nd) should be used	The extract below indicates that the *focus area selector* is a parent for part *supervisor selector,* i.e., while within the operating cycle of focus area selecting, an instance of supervisor selecting is initiated. Note the *supervisor selector* transactor has to be gray-shaded, since the *supervisor selector* is not employed by the fictitious enterprise and is initiated by the *focus area selector* that has already been identified as an environmental elementary transactor role in *Step 7* The composite transactor role student initiates the *study ethics evaluator*, using the cardinality nd	
*Step 9:* Both take turns in adding initiator links where every transactor role should be initiated at least by one other transactor role on the diagram, with the following exceptions: (1) The self-activating transactor role cannot be initiated by another transactor role, but self-activating transactor roles may initiate other transactor roles; (2) It is also possible that environmental composite transactor roles or additional elementary transactor roles (not supported by the flowchart) need to be added as initiators	In accordance with the second exception indicated in *Step 9*, a self-initiating transactor role was added, i.e., annual focus area controller, even though this role cannot be mapped to a task on the flow-chart. The cardinality of 0..* deviates from the default 1..1 cardinality for the initiating link, with the implication that one instance of annual focus area controlling may initiate zero-to-many instances of focus area selecting	
*Step 10:* Colleague duplicates green tasks that are *informational* production tasks, moving the duplicated tasks to the bottom of the drawing space. Facilitator uses the *informational* production tasks and inputs from the colleague to complete the *interstriction structure* adding *access links* when transactor roles need to access production facts from the banks of *original transaction kinds*, *multiple original transaction kinds* and *external multiple original transaction kinds*	Two green tasks, mapped from the flowchart, are both translated with a single *access link* between the focus area selector and the multiple original transaction kind *post-grad program facts*	
*Step 11:* Facilitator obtains inputs from colleague to add *wait links*, indicating that progress of an instance of one transaction kind may impede the progress of another transaction kind’s instance(s)	In the example, the progress of an instance of *proposal final evaluating* impedes (holds up) the progress of an *admitting* instance	
*Step 12:* Both validate the CSD, enquiring whether the “leaves” of the upside-down tree-structure are truly elementary. If some of the leaves can be further decomposed, i.e., they are composite (internal or environmental) transactor roles, expand the CSD by adding appropriate elementary (internal or external) transactor roles. Using the post-graduate case as an example, the initial composite transactor role named *registrar & registration payer* is a composite transactor role and may be decomposed into two elementary transactor roles, namely *registar* and *registration payer*	Step 12 of the SCM already includes the example that is exemplified in the CSD extract	

The CSD is shown on the left-hand side of Fig. 4 is similar to Fig. 1, except for the color-coded flowchart-task constructs that are added in Fig. 4. The TPT is shown in on the right-hand side of Fig. 4.

### The eSCM implementation in MURAL

**Fig. 5:**
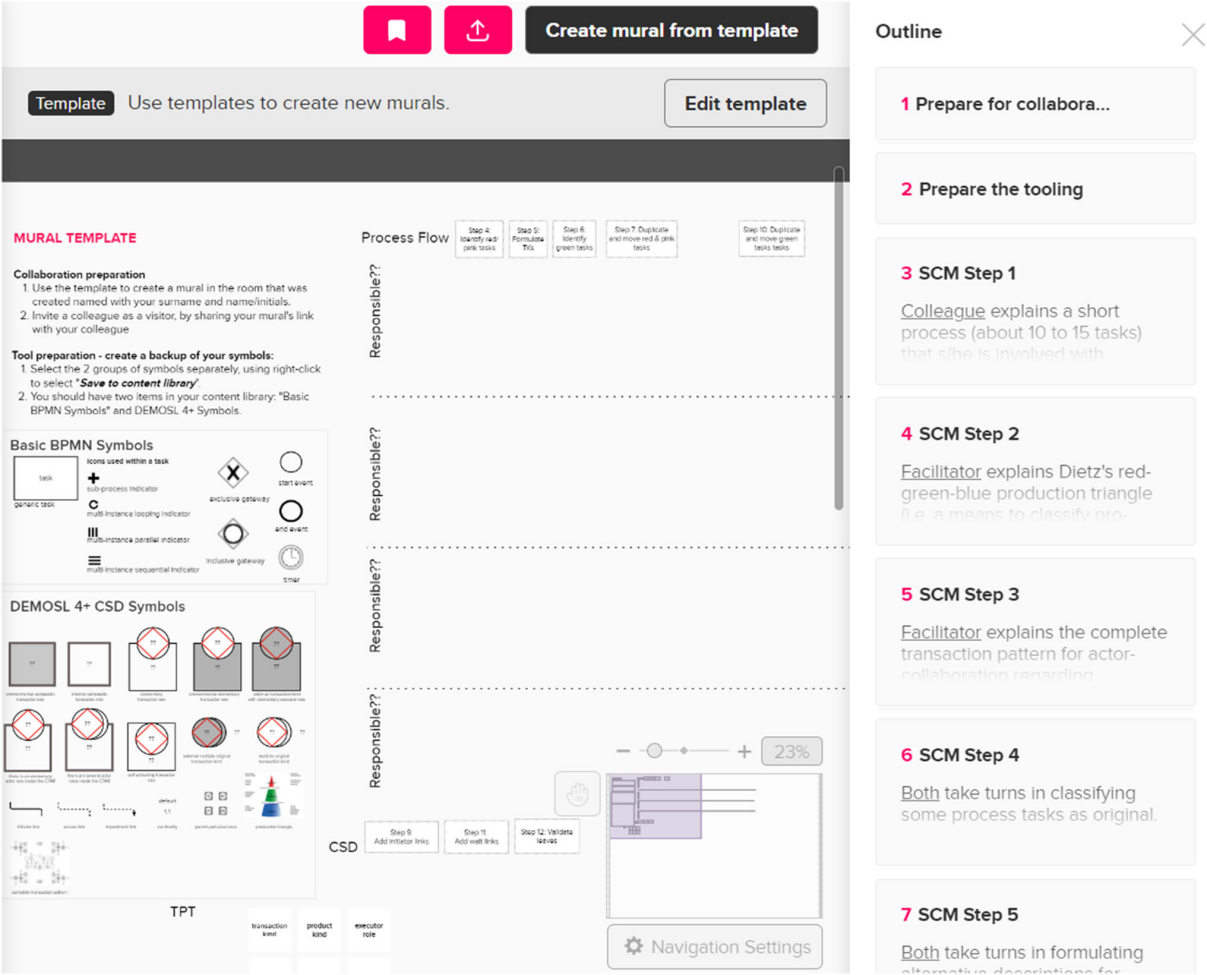
The eSCM template

Figure 5 provides an illustration of the eSCM template that was created for participants to use. On the left-hand side, two groups of symbols are available, namely *Basic BPMN Symbols* and *DEMOSL 4* + *CSD Symbols*. On the right-hand side, an *Outline* is used to provide methodical guidance in using the SCM, i.e., including 13 steps that form part of the eSCM and that are detailed in Sect. 4.2. When a user of this template uses the button *Create mural from template*, a new board (also called a mural) is created within a user-selected room. The user may invite several other users to join the board and co-model, following the steps that are listed in the *Outline*.

MURAL and the SCM template incorporates the three main characteristics defined by Stirna and Persson [22] for a participative approach, as follows: (1) The template’s Outline provided a defined *way of working* in the form of methodological steps to carry out the modeling session with explicit principles of stakeholder involvement indicated in detailed descriptions per step; (2) For each session, a facilitator leads the session, where the facilitator needs to have *knowledge* about DEMO and the facilitator has to involve a colleague that is *knowledgeable* about a particular operating context at a real-world enterprise; and (3) Each facilitator, acting as *modeling facilitator*, was responsible to guide the discussion with a colleague about using the SCM.

## Evaluation results

As indicated in Sect. 4.1, we selected MURAL as the participative modeling tool, addressing *Objective 1,* and in Sect. 4 we presented the eSCM that was designed to address *Objective 2*.

### Method

For evaluation, we involved 36 participants for applying the eSCM as facilitators, each involving a colleague from industry as a co-modeler. For *Objective 3*, evaluating whether the modeling tool *encouraged participative modeling* according to the eSCM instructions, we used a survey of 27 items (questions and probes, provided in the Appendix), voluntarily completed by 25 participants that facilitated the eSCM sessions. The survey questions in the Appendix indicate the responsibilities for data-input. Although the facilitators had to submit the survey responses, responses for two of the questions (Q21 and Q22) had to be sourced from the colleague.

Since the new modeling tool provided a feature of tracking the modeling actions of participants, we also report on the *level of participation* when participants applied the eSCM. For *Objective 4*, evaluating the *quality of the eSCM diagrams* (including the CSD), we used survey-feedback on the changes that were incorporated for the eSCM to improve the quality of the diagrams, and also evaluated the diagrams that were submitted by the 36 participants.

Providing context to the evaluation results that are presented in Sect. 5.2 to 5.4, the questions Q2–Q7, also Q10 and Q11 of the survey (see the Appendix) provided background data about the participants, i.e., the 27 group facilitators that completed the survey.

#### Participant background

Participants had an engineering background that covers multiple disciplines, i.e., industrial engineering (13 out of 25), mining (4 out of 25), metallurgy (3 out of 25), mechanical (2 out of 25), electronic (2 out of 25) and chemical (1 out of 25). Most of the participants (23 out of 25) had experience in using drawing tools or repository-based modeling tools in the past. Some of the participants used more than one tool, which included Visio (16 out of 25), Diagrams.net (9 out of 25), MURAL (2 out of 25), Miro (1 out of 25), Lucidchart (1 out of 25), MagicDraw (1 out of 25), Microsoft PowerPoint (1 out of 25), ARIS (1 out of 25) and Enterprise Architect (1 out of 25). The participants had the freedom to select a tool for verbal communication, some using *multiple* tools. Participants used MS Teams (14 out of 27), WhatsApp Calls (7 out of 27), Zoom (3 out of 27), Google Meet (2 out of 27) and a phone call (1 out of 27).

#### Structuring the results

Given the participant context, Sects. 5.2–5.4 synthesizes the results. Section 5.2 provides summative feedback on the survey results that relate to the eSCM, evaluating the eSCM’s usefulness in addressing previous deficiencies of the SCM. Section 5.3 synthesizes feedback about MURAL and its ability to facilitate *participative modeling*, also reporting on the *level of participation*, i.e., whether participants followed the participative modeling instructions of the eSCM. Section 5.4 provides summative results when evaluating the *quality of the eSCM diagrams*.

### eSCM feedback results and interpretation

The responses for questions Q8, Q9 and Q12–Q16 (see survey details in the Appendix) were used to consolidate feedback on the eSCM in addressing previous deficiencies of the SCM and whether the post-graduate case was sufficient as a demonstration case.

Answering Q8, participants had to count the number of tasks that were included in the process flowchart that had to be compiled in *Step 1* of the eSCM. A median number of 15 tasks were included, with a minimum of 10 and a maximum of 25. Answering Q9, participants also had to indicate the time duration for completing the 13 steps of the eSCM. The average time to complete was 4.7 h with a large standard deviation of 2.2 h.

As indicated in Sect. 1 the eSCM had to address some of the deficiencies that were identified when experimenting with a previous version of the eSCM. Therefore, participants had to answer questions Q12 (with Q13 to probe), Q14 (with Q15 to probe) and Q16 to evaluate whether previous deficiencies had been addressed.

**Fig. 6:**
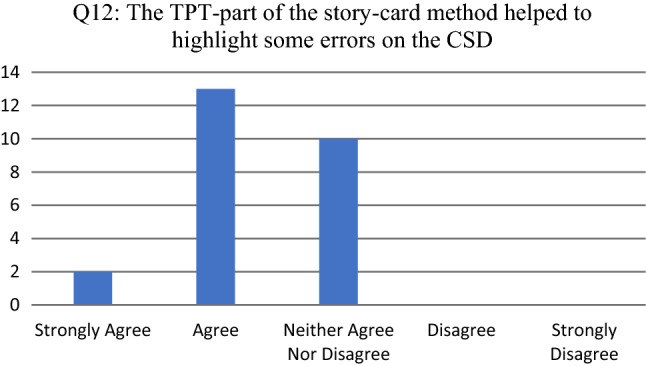
Participant responses for Q12

Results for Q12 (see Fig. 6) indicated that participants believed that the TPT-part of the SCM, a new step (Step 13) added to the previous version of the SCM (of [7]), helped to highlight some errors on the CSD. Since no participant disagree or strongly disagreed, no additional qualitative feedback was given for the probing question (Q13).

**Fig. 7:**
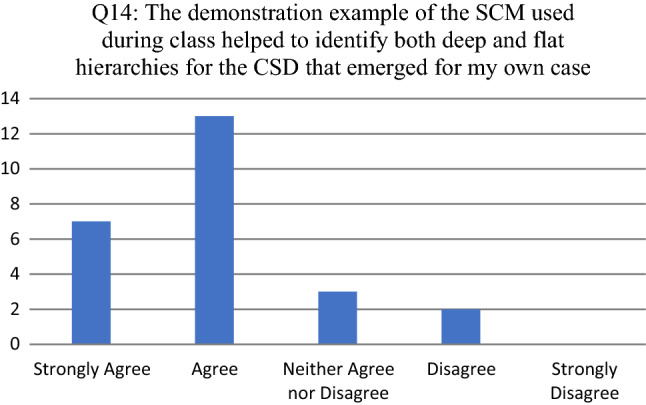
Participant responses for Q14

The results for Q14 (see Fig. 7) indicates that participants mostly agreed that the demonstration example, i.e., the post-graduate case, helped to identify both deep and flat hierarchies for the CSD. For the probing question (Q15) one of the participants indicated a lack of confidence in using the eSCM, whereas the other participant indicated that the additional example on applying the previous version of the SCM, based on the pet-sitting case (see [7]) created confusion.

Referring to Sect. 4.2 (i.e., the method steps of the eSCM), participants had an opportunity to answer an open-ended question, i.e., Q16: *If you experienced difficulties in using the story-card method, please refer back to specific steps of the story-card method exercise and motivate why you experienced difficulties.* The nine responses related to multiple eSCM steps:

- *Step 4* One respondent indicated difficulty in distinguishing the “difference between pink and red”.
- *Step 6* Three responses indicated some difficulties, i.e., *“Categorizing of the transaction types when it comes to red and green transactions can be tricky”*; *“The interpretation of the tasks as O-I-D, as well as the combination with the transaction steps. Struggled with distinctions.”*; and *“It was difficult to classify whether a task was on a green level or a light pink coordination task.”*
- *Step 9* A number of respondents referred to difficulties in identifying the *interaction structure* and cardinalities, i.e., *“struggled to identify which levels they are and how to link tasks”*; *“I realized that I had difficulties because I still don't understand when flat versus deep hierarchies apply. I think I need to go over multiple examples to gain better understanding.”*; and *“for cardinalities–it is tricky for me to understand the non-definable ones and some of the relations.”*
- *Steps 10 and 11* One respondent indicated: *“I struggled to classify the dashed connectors.”*

Some of the responses did not relate to a particular eSCM step, e.g., *“adapting my own process in terms of understanding from the class example and pet-sitting example confused me”*; and *“the only tricky part is that it took days for my colleague to understand CSD.”*

### Participative modeling results and interpretation

We evaluated participative modeling in two ways: (1) MURAL’s abilities to *encourage participative modeling*, and (2) the *level of participation* during modeling.

#### MURAL’s participative modeling abilities

**Fig. 8:**
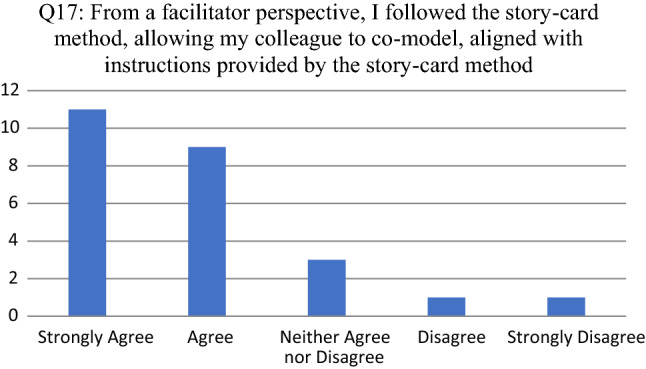
Participant responses for Q17

**Fig. 9:**
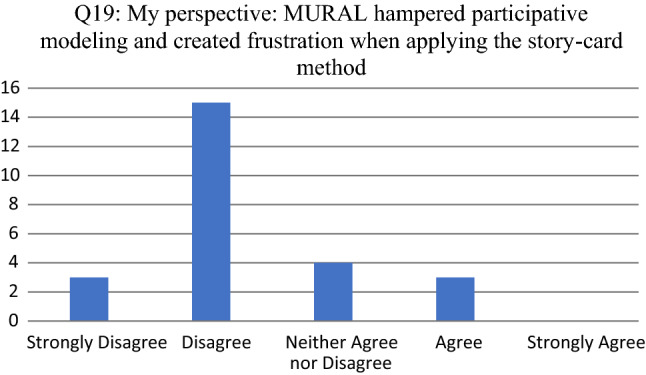
Participant responses for Q19

**Fig. 10:**
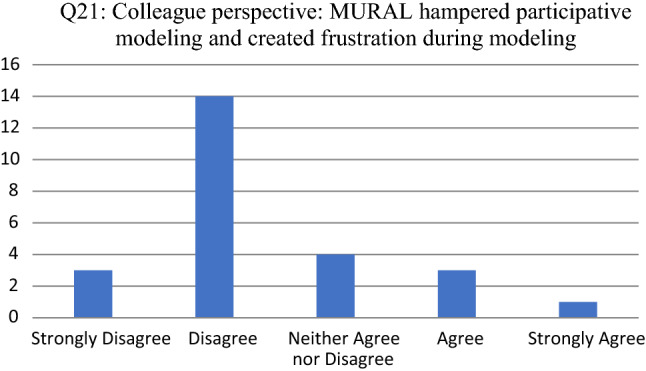
Participant responses for Q21

**Fig. 11:**
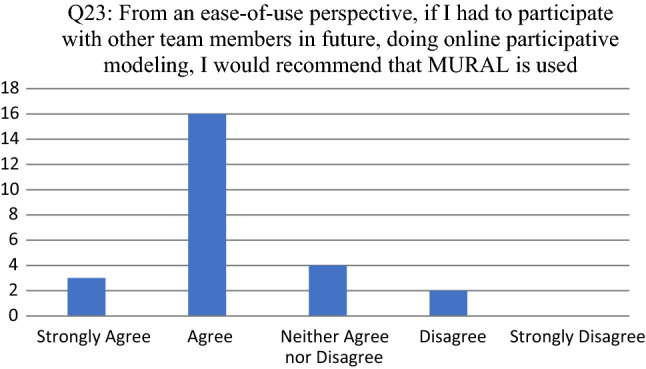
Participant responses for Q23

**Fig. 12:**
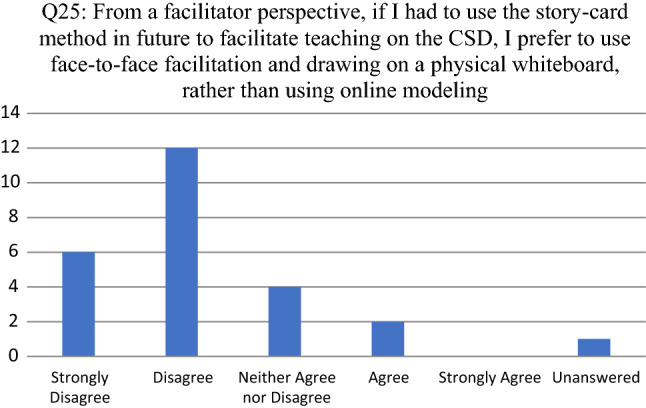
Participant responses for Q25

Opinions about the tool as enabler to facilitate participative modeling are consolidated in Figs. 8, 9, 10, 11 and 12. For each of the reported survey questions (Q17, Q19, Q21, Q23 and Q25), probing questions (Q18, Q20, Q22, Q24 and Q26) were used, encouraging participants to further motivate if they deviated from an expected opinion.

For Q17 (see Fig. 8) the participants that disagreed or strongly disagreed indicated that their colleague that co-modeled the operating context was not from an engineering background and had difficulties in understanding the concepts used in the eSCM.

For Q19 (see Fig. 9) the 3 participants that agreed, indicating that (1) some functionalities in MURAL (e.g., ctrl-c) did not work; (2) the “undo” function did not always work; and (3) the exports from MURAL are not readable.

For Q21 (see Fig. 10), the 3 participants that agreed provided additional motivation, indicating that (1) the colleague found it challenging to complete the process flow, since MURAL is not as user friendly as Visio; (2) the colleague did not understand the new concepts and did not appreciate the value of the new modeling language; and (3) MURAL would often refresh automatically causing distraction and time loss.

For Q23 (see Fig. 11) 1 of the 2 participants that disagreed provided additional motivation, indicating that many companies already have modeling tools that are more self-explanatory than MURAL.

For Q25 (see Fig. 12) the 2 participants that agreed indicated that (1) people engage better with face-to-face facilitation and the first session should be using a whiteboard followed by more online examples; and (2) zooming in and out on the SCM caused frustrations with navigating.

Q27 was in the form of an open-ended question: *In terms of the MURAL tooling, you or your colleague may have experienced some frustrations related to the tool functionality. Please elaborate on these frustrations.* The following categories or themes emerged from the responses. For each identified category we quote all of the qualitative responses that are associated with the identified category:

- The ‘undo’ function is not working properly, e.g., *“MURAL has issues tracking changes chronologically, for example at times we would use the undo function only to revert to a ‘previous’ instance that didn't actually exist”* and *“The undo function would also often not work”.*
- Lack of auto-alignment, e.g., *“my colleague wanted to create a neat flow chart from the beginning but constantly had to re-do portions of the process flow.”*
- Editing frustrations, e.g., not being able to *“edit the style of multiple text items simultaneously”* and *“In some cases one person had a block still selected while the other person was trying to edit the text.”*
- Using an “area” as a container for constructs created problems, e.g., not always being able to edit constructs inside an area, and *“When adding an area, at times this would disorient my flow chart.”*
- Automatic sticky note addition with double-click, e.g., a double-click on an editable construct will add a sticky note construct, instead of editing the text of the editable construct.
- Auto-refresh problems, e.g., “*Some of the icons (Gateways and Timer) also did not show, it was just a gray block, I needed to refresh the page a couple of times before it returned to normal”* and *“MURAL would often refresh causing distraction and time loss.”*
- Activity tracking “*did not work properly*” and *“My activity tracking was cleared out.”*
- Work loss due to connection problems, e.g., *“My colleague lost connection, but did not realize it until five tasks later (I noticed but I first thought it was from my side), and then he had to reconnect. When he reconnected, the tasks disappeared and he had to re-draw all of them again.”*
- MURAL should have the ability to verbally communicate while modeling, e.g., *“It would be easier if MURAL itself came with the option to communicate verbally while collaborating instead of having to use WhatsApp.”*

Some of the open-ended responses were positive, even though the Q27 requested feedback regarding *tool frustrations*, e.g., *“My colleague and I both agreed that the MURAL tooling was efficient and easy to use*” and “*I found the tool to be very user friendly—I did not experience major difficulties.”*

#### Actual level of participation

MURAL offers an activity tracking tool that was useful to determine whether participants engaged with a colleague, using the eSCM as intended, where both participants had to apply the co-modeling instructions of the eSCM. Two of the participants indicated that they have lost the detailed history of their interactive modeling activities. Although we investigated various possible reasons, e.g., the duration from creation date to last-editing date, connection problems, and the total number of editing activities, we could not confirm any of these possibilities. Furthermore, we investigated the 36 individual murals to determine if the entire history of the mural (from creation date) reflected in MURAL and found that only 21 of the murals were traceable from the creation date. A visual inspection of the activity history was required to determine whether participants indeed followed the prescribed participation instructions that were indicated for some of the eSCM steps. Although *Step 2*, *Step 3* and *Step 8* of the eSCM required explanation by the facilitator alone, whereas *Step 12* is a validation step that may not necessarily require additional modeling, the remaining steps, included participative modeling with the following dedicated modeling responsibilities:

- *Step 1* Colleague maps out process
- *Step 4* Both classify process tasks for o-level
- *Step 5* Both formulate alternative descriptions
- *Step 6* Both analyze tasks for i-level
- *Step 7* Facilitator duplicates red and light-red tasks
- *Step 9* Both take turns to model initiator links
- *Step 10a* Colleague duplicates green tasks
- *Step 10b* Facilitator models access links
- *Step 11* Facilitator models wait links
- *Step 13* Both take turns in TPT

**Fig. 13:**
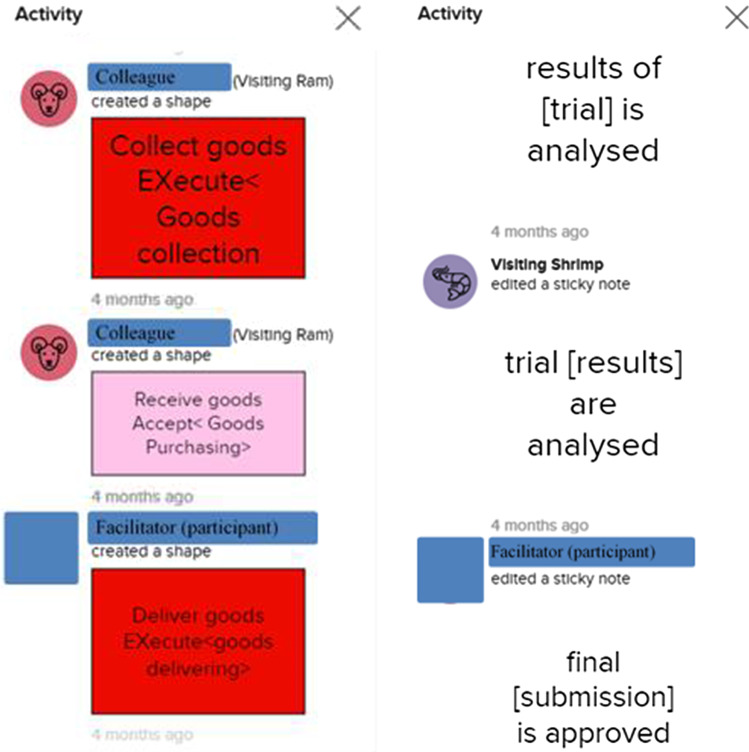
Activity log extracts from participants

For demonstration purposes, extracts of the activity logs for 2 randomly selected participants are shown in Fig. 13. The activity log on the left-hand side demonstrates method *Steps 4* and *5*, whereas the activity log on the right-hand side demonstrates method *Step 13.* For the examples extracted, both participants adhered to the modeling responsibilities of both for the relevant method steps, i.e., both the colleague and the facilitator participated during the modeling steps. The identities of the participants and their colleagues were concealed using blue blocks.

**Fig. 14:**
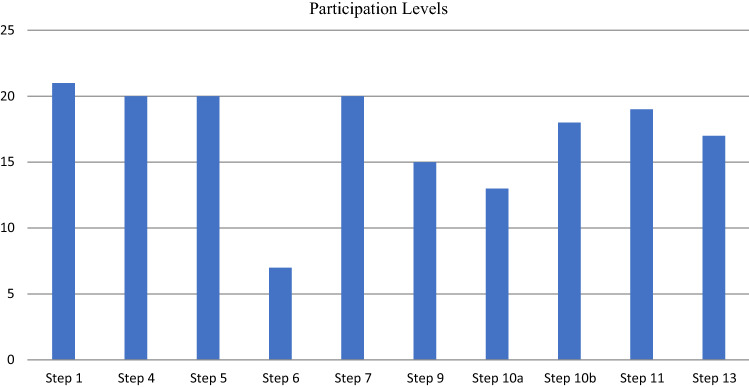
Activity tracking results to indicate participation levels

A Microsoft Excel spreadsheet was used to analyze the results obtained from visually inspecting the activity logs, of which extracts are shown in Fig. 13. For each of modeling-related eSCM steps, a column was created indicating with a “0” or “1” whether the modeling participant adhered to the indicated modeling responsibilities. The “0” indicated that the step *was not executed* by the step-required role(s) and “1” indicated that the step *was executed* by the step-required role(s). Counting the number of participants (out of 21 murals), adhering to the step-related responsibilities, the participation results are synthesized in Fig. 14. The results indicate *high participation levels*, i.e., participants co-modeled according to the allocated responsibilities, for most of the modeling-related eSCM steps.

Referring to Fig. 14, we highlight three steps with lower participation. *Step 6*, where both the facilitator and colleague had to identify *i*-level tasks, is problematic, since the facilitator performed the identification, rather than the colleague. For *Step 9* we expected that the participation pairs would have difficulty in identifying initiation links and the results confirmed that the facilitator controlled this step, rather than the colleague. *Step 10a* had to be performed by the colleague, but for some participative sessions, the facilitator performed *Step 10a*, which may be due to the fact that the facilitator wanted to expedite the session.

### Diagram quality results and interpretation

The 36 participants that applied the eSCM had to submit four *diagram types* (A, B, C and D) as evidence for implementing the 13 method steps, as indicated in Table 4’s first column. Since the SCM was presented at the Enterprise Engineering Working Conference of 2021 [7], experts on DEMO aspect models and diagram types, provided feedback about the granularity of the evaluation criteria, suggesting further refinement in evaluating the diagram types. The feedback was incorporated in preparing the evaluation criteria presented in Table 4.

**Table 4:** Evaluation criteria and descriptive statistics for evaluation results

Diagram types	Evaluation criteria				Evaluation results	
	novice	limited understanding	Some understanding	Full understanding (maximum points)	Average (%)	Std dev (points)
A: Diagram similar to Fig. 2 (*Step 1*)	0 points: Section empty. AND/OR Image not readable. AND/OR Less than 10 tasks. AND/OR more than 1 error in task phrasing	0 points: See Novice	0.5 Points: 1 error, i.e., one of the tasks not phrased using the "verb + noun" standard	1 Points: Followed all instructions for SCM Step 1	91.67	0.108
B: Diagram similar to Fig. 3 (*Steps 4 to 6*)	0 Points: Section empty. AND/OR Diagram not readable. AND/OR Not composed using the MURAL SCM template	1 Points: More than 1 error	2 Points: 1 error in terms of red/pink/green classification OR 1 error in alternative phrasing	3 Points: Followed all instructions for SCM Steps 4–6. No errors	61.11	0.775
C: Diagram similar to Fig. 4’s left-hand side (*Steps 7 to 12*)	0 Points: Section empty. AND/OR Diagram not readable. AND/OR No flowchart tasks are mapped to TARs. AND/OR Not composed using the MURAL SCM template	1 Points: More than 1 CSD error	2 Points: 1 error in terms of CSD errors (see class notes)	3 Points: Followed all instructions for SCM Steps 7–12 to generate a valid CSD according to the DEMOSL 4 + standard. No errors	50.93	0.609
D: Diagram similar to Fig. 4’s right-hand side (*Step 13*)	0 Points: Section empty. AND/OR TPT not readable. AND/OR Not composed using the MURAL SCM template	1 Points: More than 1 TPT error	2 Points: 1 error in terms of TPT errors (see class notes)	3 Points: TPT is 100% aligned to the CSD, no errors	53.70	0.766

The second column of Table 4 provides *evaluation criteria* per diagram type, and grading per criterion. Since the diagram types required different skill levels, *diagram type A* could earn a maximum of 1 point, whereas the other three diagram types could earn a maximum of 3 points. For *diagram type A*, the colleague had to map out a process using a flowchart, whereas diagram types B, C and D, were more complex, i.e., they required execution of multiple eSCM steps, with additional analysis and deliberation between participants. We needed more granularity in grading diagram types B, C and D, with a maximum score of 3 points per diagram.

The descriptive statistics for the evaluation results are summarized in the third column, Table 4, i.e., *evaluation results*, explained further in the subsequent paragraphs.

#### Diagram type A

The results indicate that participants scored an average 91.67% for completing diagram type A, i.e., following the flowchart-compiling step of the eSCM. Only one error-type could exist, as indicated in Table 4, namely that one of the tasks in the flowchart is not phrased using the "verb + noun" standard.

#### Diagram type B

The average score for diagram type B was 61.11%, highlighted existence of two error-types: (1) Incorrect red/pink/green classification of tasks on the flowchart, and (2) Incorrect re-phrase of tasks according to the alternative phrasing for flowchart tasks that relate to the complete transaction pattern.

#### Diagram type C

Participants scored an average of 50.92% for diagram type C indicating that the CSDs were faulty in terms of different error-types. With reference to Table 3, indicating how *Step 7* to *Step 12* contribute toward the construction of the CSD, multiple error-types exist. As an example, for *Step 9*, the eSCM indicates “*The self-activating transactor role cannot be initiated by another transactor role, but self-activating transactor roles may initiate other transactor roles.*” A common error-type is that the CSDs contained self-activating transactor roles that were initiated by another transactor role.

#### Diagram type D

An average score of 53.70% was obtained for diagram type D, with two prominent error-types: (1) The product kind has no variable(s) or appropriate variable(s) to differentiate between two instances of the product kind, and (2) the product kind includes a variable that is not periodic of nature, whereas the CSD indicates that the transactor role, associated with the product kind, is self-activating.

Lower averages for diagram types B, C and D corroborate with the qualitative feedback from participants regarding the difficulty of performing steps 4, 6 and steps 9 to 11, indicated in Sect. 5.2. Even though the eSCM added Step 13 to the original SCM in [7] to improve the quality of the CSD and participants indicated that the TPT helped to highlight some errors on the CSD (see Sect. 5.2), i.e., to increase the average score for diagram type C, the results indicate that low-quality CSDs are still produced.

## Discussion, limitations and future research

A study that focused on the development and evaluation of an online-adapted version of the SCM [7], indicated two main concerns with the SCM: (1) The previous modeling software does not encourage *active participation* during modeling; and (2) The low quality of the resulting cooperation structure diagram (CSD) and hence its derived enterprise operation taxonomy. In addressing these main concerns, we applied DSR to further evolve the SCM into an eSCM, highlighting 4 main objectives for our study.

### Discussion

This section presents a synthesis of our findings in terms of the four main objectives of the study, presented in Sect. 2.2.

*Objective 1* As indicated in Sect. 4.1, we selected MURAL as the participative modeling tool, addressing *Objective 1*. We believe that the main features that we identified during an explorative approach (see Table 1), experimenting with two participative modeling tools, i.e., Miro’s and MURAL’s Education Plan options, are also useful to other researchers when they need to compare multiple tools within their own participative modeling context.

*Objective 2* In Sect. 4 we presented the eSCM that was designed to address *Objective 2,* applying principles from participative enterprise modeling (PEM). From a design perspective, MURAL allowed us to create an *eSCM template* that supported both diagram construction via *pre-designed symbols*, as well as *method guidance*, to facilitate PEM, as follows. The study participants acted as session facilitators, each creating a new mural, based on the eSCM template. When facilitators invited co-modelers to their mural, their co-modelers had access to two groups of symbols, namely *Basic BPMN Symbols* and *DEMOSL 4* + *CSD Symbols*. In addition, the individual eSCM steps, included in an *Outline* part of the template, provided *method guidance* (as detailed in Sect. 4.1), i.e., including the 13 steps with modeling responsibilities (facilitator, colleague or both) to *encourage participative modeling*.

*Objective 3* For *Objective 3*, evaluating whether the modeling tool *encouraged participative modeling* according to the eSCM instructions, we used a survey, voluntarily completed by 25 participants. Since the new modeling tool provided a feature of tracking the modeling actions of mural-participants, we also reported on the *level of participation* when participants, together with their colleagues, applied the eSCM. The survey results indicated that participants had a positive experience when they used MURAL in combination with the eSCM, i.e., that MURAL *encouraged participative modeling*. Some participants also experienced frustrations with some of MURAL’s functions, including: (1) the “undo” function, (2) auto-alignment, (3) editing, (4) using the “area” construct, (5) automatic sticky note addition with double-click, (6) auto-refresh problems, (7) activity tracking not working properly, (8) work loss due to connection problems, and (9) the inability to communicate verbally via MURAL. MURAL continuously improve their product, based on error-reporting and requirements from the end user community, also communicating their product updates via their website: https://www.mural.co/changelog.

The activity tracking results also indicated *high levels of participation* in accordance with the dedicated modeling responsibilities of the eSCM. Steps, where 6 or more of the participant-pairs deviated from the modeling instructions, include Step 6 (Both analyze tasks for i-level), Step 9 (Both take turns to model initiator links) and Step 10a (Colleague duplicates green tasks). The detailed analysis or the activity histories indicated that the facilitator would take control of the modeling, which may be due to several reasons. We believe that the facilitator might be expediting the session, since the average duration of completing an eSCM session, is 4.7 h. It is also possible that the facilitator had difficulty to impart knowledge on DEMO, since survey-feedback, regarding problematic eSCM steps, also included the same steps (Steps 6, 9 and 10), as discussed in the next paragraph.

*Objective 4* For *Objective 4*, evaluating the *quality of the eSCM diagrams* (including the CSD), we used survey-feedback on the changes that were incorporated for the eSCM to improve the quality of the diagrams. In addition, using evaluation criteria, we evaluated the quality of the diagrams that were submitted by the 36 participants.

We expected that the TPT would improve the quality of the CSD. For *Step 13* of the eSCM, the participant-pair had to validate the CSD by compiling a TPT. Each of the transactors on the CSD are mapped as executor roles on the TPT. For each executor role, a corresponding transaction kind and product kind should be added. Although the terminology associated with the TPT differs from that of the better-known Create-Read-Update-Delete (CRUD) matrix found in [35] and [36], the TPT is similar to the CRUD matrix in mapping the process-logic to data/fact-logic. Whereas the data-to-process CRUD matrix evaluates completeness of *process requirements* and *data requirements* when information system *processes* are mapped to *data* elements in creating/reading/updating/deleting data in a database, the TPT also maps process logic, consolidated into original *transaction kinds*, to original *product kinds*. Identifying an appropriate *product kind* per *transaction kind* assists in validating that every transaction kind’s associated transactor on the CSD, is indeed an original transaction kind. It is possible to incorrectly classify flowchart tasks, using red or pink color-coding, as original transactions kinds during Step 4 of the eSCM. The TPT allows for additional validation of the CSD, correcting three types of errors:

- *Type 1* Remove a transactor that is a duplicate of another transactor when both transactors produce the same product kind.
- *Type 2* Replace a non-self-activating transactor by a self-activating transactor, removing the initiation link, if the product kind includes a periodic variable. Alternatively, change a self-activating transactor into a non-self-activating transactor if the product kind does not include a periodic variable, also adding an appropriate initiation link.
- *Type 3* Remove documental or informational transactors, since the transactor does not produce a new/original production fact.

Using Fig. 4’s TPT, depicted on the right-hand side of the diagram, we provide an example per error-type to demonstrate the error-detecting abilities of the TPT:

- *Type 1* If the TPT already included the transaction kind *focus area controlling* with product kind *focus area control for [year] is completed*, and a second transaction kind *research theme controlling* with product kind *focus area control for [year] is completed*, the transactor *research theme controller* has to be removed.
- *Type 2* If the TPT indicates for transaction kind *focus area controlling* the product kind *focus area control for [year] is completed*, and the CSD included an elementary transactor that is initiated by another transactor, the interaction structure of is incorrect. The initiation link has to be removed and the elementary transactor has to be replaced by a self-activating transactor.
- *Type 3* If the TPT included the transaction kind *focus area documenting* with product kind *the [focus area] facts are shared on the web site*, the documental transactor has to be removed from the CSD.

The survey-feedback indicated that participants were positive about the addition of Step 13, i.e., none of the participants disagreed with the statement that *“the TPT-part of the story-card method helped to highlight some errors on the CSD*.*”* Also, participants were mostly positive (strongly agreed or agreed) that the demonstration example of the SCM, i.e., the post-graduate demonstration case, *“helped to identify both deep and flat hierarchies for the CSD that emerged”* for their own case. Yet, participants still experienced numerous difficulties when applying the eSCM. The open-ended responses indicated that participants still struggled with Step 4 (classifying process tasks as original), Step 6 (identifying informational tasks), Step 9 (adding initiation links for the CSD), Step 10 (converting informational tasks to access links on the CSD) and Step 11 (adding wait links to the CSD). Three of the five problematic steps, relate to *constructing the CSD*. On evaluating the quality of the diagram types that emerged from the 36 participant-pairs, the results indicated that diagram type C, i.e., the CSDs, scored the lowest average score of all the diagram types, i.e., 50.93%.

### Limitations and future work

We now discuss the limitations and future work in terms of the two main concerns that our study had to address, namely (1) participation during modeling; and (2) the low quality of the resulting cooperation structure diagram (CSD).

#### Participation during modeling

Our results indicated that the eSCM as implemented via MURAL encouraged active participation of participant-pairs. Since MURAL’s activity tracking functionality did not work properly in displaying the full history of all participant-pairs, we could only use 25 out of the 36 murals to analyze the *level of participation* on the murals. The sample of 25 murals was sufficient to identify the problematic eSCM steps. The subsequent paragraphs present some of the limitations of our study and ideas for future work. We present the limitations in order of priority, starting with the high-priority items. The first two limitations may require further changes to the eSCM, whereas the third and fourth limitation are tool-related.

*The participant-pairs limitation* Although we followed principles, guidelines and structuring that would encourage participation, when developing the eSCM, the eSCM only facilitates a PEM session between 2 participants, i.e., *participant-pairs*. Experimenting with participant-pairs created consistency in the group size for the 36 groups, reducing the number of variables that may affect the levels of participation. When the eSCM is used to co-model an operating context within an agile software development team with five of more team members, the non-facilitator members of the team, will be replacing the existing colleague role in the eSCM. Based on the team members, their roles and expertise, the facilitator may have to edit the *Outline* part of the eSCM, re-allocating the modeling activities where the colleague was involved, to different team members. For future work, we suggest that a demonstration case that we now refer to as *case-future*, is developed to demonstrate the re-allocation of modeling activities to different team members, and the adaptation of the *Outline* part.

* The limitation of the eSCM not facilitating co-modeler context* As indicated by Gutschmidt et al. [37], a prerequisite for constructive participation is that participants need to know one another. Our experiment mitigated the risk of hampered participation due to limited relationship-building, providing the facilitator of the participative session the freedom to select a colleague. We acknowledge that a free selection of participants will not be the case in real-world enterprise modeling scenarios. The eSCM may have to be supplemented with *an introductory phase* where participants share their project context and existing roles at an enterprise. For future work, a demonstration case, i.e., *case-future*, should demonstrate how different participants share their *project context* and existing *roles*, e.g., a participant may indicate as *project context* that s/he is working on a software development project that automates the workflow of processing requests for examination concessions, where the participant has the *role* of systems analyst.

*The limitation of not gathering data about the technology used* Our results indicated that *participant-pairs* experience frustrations with nine different MURAL functions. Using the tool for a larger group of participants, e.g., members of an agile software development team, may surface additional problems and frustrations with the tooling. We have not considered the hardware that participants used, which may have affected their overall experience, especially regarding the output devices and the availability of a second monitor. Our study was not prescriptive in using a synchronous voice communication tool, such as Zoom. Rather, we allowed participants to select a tool, decreasing the learning curve of introducing multiple tools. Some of the participative modeling tools, such as FigShare, already include a chat communication function. Additional experimentation with such a tool with a built-in chat function is suggested for future research.

*Limited measuring of tool ease-of-modeling and usability* The researchers experimented with multiple participative modeling tools in a separate experiment to ensure ease-of-modeling and that the selected tool was useful in supporting the eSCM, as discussed in Sect. 4.1. Some of the questions in the survey, i.e., questions Q17 to A27 in the Appendix, also evaluated MURAL’s participative abilities from a participant’s perspective. This study focused on selecting the most appropriate tool to support the eSCM, encouraging co-modeling. Therefore, the general usability of MURAL, using a standardized usability survey, such as the Software Usability Measurement Inventory (SUMI) of Kirakowski and Corbett [38] that includes 50 usability statements, was not used. Adding an additional 50 usability statements to the existing survey of 27 questions would over-burden the participant that already applied the eSCM and completed the survey. However, we believe that a usability assessment of MURAL will provide additional confidence, from a participant’s perspective, regarding the general usability of the tool. For future work, a separate tool usability study is suggested, where different usability theories are considered, such as the Unified Theory of Acceptance and Use of Technology (UTAUT) [39], to develop an appropriate questionnaire to assess MURAL’s usability.

#### Quality of the cooperation structure diagram

Although the eSCM already extended the original steps associated with the SCM of [7], adding a post-graduate demonstration case, the quality of the CSDs are still unacceptably low. The participants, i.e., the facilitators of the eSCM sessions, received 4 h of training on aspect model theory, 4 h of training on the four aspect models, applied to a comprehensive fictitious college case, and a 4 h-demonstration of the eSCM, using the post-graduate case. In addition, participants had access to all the cases presented in [13]. Even though the eSCM assists the facilitator, helping with *knowledge transfer* of some aspect model concepts to a co-modeler, i.e., the colleague, using an operating context that is familiar to the colleague, we believe that the facilitator needs much more experience and exposure to different case studies to effectively guide an eSCM session. The eSCM, packaged within MURAL, has the potential of involving novice co-modelers if the eSCM-facilitator is well-trained.

In addressing the quality of the CSD we believe that *additional training* is needed. The evaluation results of the four diagram types, already indicated common errors that should also be pre-empted during training, e.g., emphasizing during training that the complete transaction pattern is applicable to both *informational* transaction kinds and *original* transaction kinds. We believe that *more than one learning cycle*, based on different operating contexts, may be required to ensure that a facilitator develops adequate expertise on aspect model concepts. For future work, we plan to experiment with multiple learning cycles when facilitators-in-training need to compile a CSD from different operating contexts. For each learning cycle, a facilitator has to receive feedback on the quality of the CSD, highlighting the errors. A facilitator should reach an acceptable level of expertise to act as a CSD modeling expert, prior to facilitating an eSCM session to co-model a high-quality CSD that is useful as an *enterprise operation taxonomy* for an agile software development team.

## 7 Appendix: Survey

The survey questions were used to obtain data to provide eSCM feedback (refer to Sect. 5.2) and feedback about participative modeling (refer to Sect. 5.3).

### 7.1 Ethical clearance question

Q1: By selecting the "Yes" option I hereby voluntarily grant my permission for participation in this anonymous survey. The nature and the objective of this research have been explained to me and I understand it. I understand my right to choose whether to participate in the research project and that the information provided will be handled confidentially. I am aware that the results of the survey may be used for academic publication.

### 7.2 Participant background questions

Q2: Indicate your existing role at the enterprise. Examples of roles include: Lecturer assistant, Business or systems analyst or consultant (also called improvement specialist or consultant), Supply chain analyst or consultant, Logistics specialist, Warehouse planner OR product replenishment planner, Software or software solution analyst or developer etc.

Q3: Indicate the MAIN type of industry where you are currently employed.

Full time students: Please indicate "Full time student" at the bottom of the list. The list includes: Aerospace and defense manufacturing, Automotive, Chemicals, Construction/engineering, Consumer sector or consumer packaged goods, Education, Financial services, Health care, High technology manufacturing, Independent software vendor, Industrial manufacturing, Life sciences (biotech, pharmaceuticals), Logistics consulting, Media and entertainment, Mining, Oil and gas, Outsourcing, Professional consulting services, Research, Retail / wholesale / distribution, Telecommunications, Travel and transportation, Utilities (electric, gas, sanitation, water), FULL TIME STUDENT, OTHER.

Q4: If you selected "OTHER" in the previous question, please specify the type of industry for your current employment.

Q5: Please specify your tertiary qualification, e.g., BEng (Industrial).

Q6: Please investigate whether your enterprise is currently using modeling tools to represent process/activity logic at the enterprise. You may select more than one option, if your enterprise is using multiple tools.

ARIS (by Software AG), CaseWise Modeler (by Ervin Inc.), Enterprise Architect (by Sparx), Open Modeling (open source initiative), uRequire Studio (by uSoft), Visio (by Microsoft), ABACUS (by Avolution), Lucidchart.

Diagrams.net (previously Draw.io), Miro, Mural, Figma/FigJam, Other.

Q7: If you answered "other" in the previous question, please indicate other modeling tools currently used for process/activity related modeling.

Q10: Please indicate the type of verbal online communication technology/tool that you used to facilitate the discussion: Google Meet, MS Teams, Zoom, Blackboard Collaborate, Skype, Bluejeans, Other.

Q11: If you indicated "other" in the previous question, specify the "other" verbal online communication tool that you used to facilitate the discussion.

### 7.3 Questions on using the eSCM to encourage participative modeling

Q8: Count the number of tasks in your "story" (note that a decision-diamond in itself is not a task).

Q9: How many minutes did you spend on this exercise, i.e., performing all steps of the story-card method exercise?

Q12: The TPT-part of the story-card method helped to highlight some errors on the CSD: Strongly Agree, Agree, Neither Agree nor Disagree, Disagree, Strongly Disagree.

Q13: If you "disagreed" or "strongly disagreed" in the previous question, provide a motivation.

Q14: The demonstration example of the SCM used during class helped to identify both deep and flat hierarchies for the CSD that emerged for my own case: Strongly Agree, Agree, Neither Agree nor Disagree, Disagree, Strongly Disagree.

Q15: If you "disagreed" or "strongly disagreed" in the previous question, provide a motivation.

Q16: If you experienced difficulties in using the story-card method, please refer back to specific steps of the story-card method exercise and motivate why you experienced difficulties.

### 7.4 Questions on the participative modeling and the abilities of MURAL

Q17: From a facilitator perspective, I followed the story-card method, allowing my colleague to co-model, aligned with instructions provided by the story-card method: Strongly Agree, Agree, Neither Agree nor Disagree, Disagree, Strongly Disagree.

Q18: If you "disagreed" or "strongly disagreed" in the previous question, provide a motivation.

Q19: My perspective: MURAL hampered participative modeling and created frustration when applying the story-card method: Strongly Agree, Agree, Neither Agree nor Disagree, Disagree, Strongly Disagree.

Q20: If you "agreed" or "strongly agreed" in the previous question, provide a motivation.

Q21: Colleague perspective: MURAL hampered participative modeling and created frustration during modeling: Strongly Agree, Agree, Neither Agree nor Disagree, Disagree, Strongly Disagree.

Q22: If you "agreed" or "strongly agreed" in the previous question, provide a motivation.

Q23: From an ease-of-use perspective, if I had to participate with other team members in future, doing online participative modeling, I would recommend that MURAL is used: Strongly Agree, Agree, Neither Agree nor Disagree, Disagree, Strongly Disagree.

Q24: If you "disagreed" or "strongly disagreed" in the previous question, provide a motivation.

Q25: From a facilitator perspective, if I had to use the story-card method in future to facilitate teaching on the CSD, I prefer to use face-to-face facilitation and drawing on a physical whiteboard, rather than using online modeling: Strongly Agree, Agree, Neither Agree nor Disagree, Disagree, Strongly Disagree.

Q26: If you "agreed" or "strongly agreed" in the previous question, provide a motivation.

Q27: In terms of the MURAL tooling, you or your colleague may have experienced some frustrations related to the tool functionality. Please elaborate on these frustrations.
